# Antioxidant and Antimicrobial Properties of Mung Bean Phyto-Film Combined with Longkong Pericarp Extract and Sonication

**DOI:** 10.3390/membranes12040379

**Published:** 2022-03-30

**Authors:** Ittiporn Keawpeng, Balaji Paulraj, Karthikeyan Venkatachalam

**Affiliations:** 1 Faculty of Agricultural Technology, Songkhla Rajabhat University, Muang, Songkhla 90000, Thailand; ittiporn.ke@skru.ac.th; 2 PG and Research Centre in Biotechnology, MGR College, Hosur 635130, Tamil Nadu, India; balaji_paulraj@yahoo.com; 3 Faculty of Innovative Agriculture and Fishery Establishment Project, Prince of Songkla University Surat Thani Campus, Makham Tia, Mueang, Surat Thani 84000, Thailand

**Keywords:** mung bean flour, longkong pericarp extract, sonication, phyto-films, antioxidant and antimicrobial properties

## Abstract

Mung bean (*Vigna radiata*) flour serves as an excellent biopolymer and a potential material for producing antioxidant and antimicrobial phyto-films. In addition to mung bean flour, this study also combined the longkong (Aglaia dookkoo Griff.) pericarp extract (LPE, 1.5%) and ultrasonication process (0 (C1), 2 (T1), 4 (T2), 6 (T3), 8 (T4), and 10 (T5) min, sonicated at 25 kHz, 100% amplitude) in film emulsion production to improve the antioxidant and antimicrobial efficiency in the phyto-films. This study showed that sonication increased the phyto-films’ color into more lightness and yellowness, and the intensity of the color changes was in accordance with the increased sonication time. Alternatively, the thickness, water vapor permeability, and solubility of the films were adversely affected by extended sonication. In addition, elongation at break and tensile strength increased while the Young modulus decreased in the phyto-films with the extended sonication. Furthermore, the droplet size and polydispersity index of the phyto-films decreased with extended sonication. Conversely, the zeta potential of the film tended to increase with the treatments. Furthermore, phytochemicals such as total phenolic content and total flavonoid contents, and the radical scavenging ability of phyto-films against the DPPH radical, ABTS radical, superoxide radical, hydroxyl radical, and ferrous chelating activity, were significantly higher, and they were steadily increased in the films with the extended sonication time. Furthermore, the phyto-films showed a significant control against Gram (-) pathogens, followed by Gram (+) pathogens. A higher inhibitory effect was noted against L. monocytogens, followed by *S. aureus* and *B. subtilis*. Similarly, the phyto-films also significantly inhibited the Gram (-) pathogens, and significant control was noted against *C. jejuni*, followed by *E. coli* and *P. aeruginosa*. Regardless of the mung bean flour, this study found that longkong pericarp extract and the sonication process could also effectively be used in the film emulsions to enhance the efficiency of the antioxidant and antimicrobial properties of phyto-films.

## 1. Introduction

Leguminous seeds are the richest and cheapest phyto-macromolecules, particularly proteins and carbohydrates [1]. Despite the macromolecules’ abundance, the quality of these components is usually inadequate due to the shortage of sulfur-containing amino acids, the presence of anti-physiological properties, and the occurrence of various toxic factors due to insufficient processing. Furthermore, leguminous seeds are rich in phytochemicals and functional properties [2]. Mung bean (*Vigna radiata* (L.) Wilczek), also known as green gram, is widely cultivated in Asia and abundantly used as a staple food or a medicinal ingredient in South Asia and South-East Asia countries [3]. Mung bean is one of the a few legumes that is composed of unique macro and micronutrients as well as numerous phytochemicals, including phenolic acids, flavonoids, sterols, terpenes, alkaloids, and organic acids. In addition, the mung bean possesses numerous functional properties, including antioxidant, antimicrobial, antilipidemic, anticarcinogenic, antitumor, and detoxification properties [4]. Recent advancements in applied phytotechnology provide vast opportunities to incorporate plant-based materials into value-added products and prepare functional materials or tailor them into pharmacological materials [5]. The majority of these products are water-soluble or biodegradable phyto-films, mainly composed of biopolymers (proteins, polysaccharides, and lipids) and hydrocolloids (carrageenan) gums), which are renewable, inexpensive, and environmentally friendly composites [6]. Furthermore, utilization of plant extracts combined with the phyto-film has shown good effects, as they could increase the stability of the stored product and could provide a barrier against spoilage and pathogenic microorganisms. They could also control the biochemical degradation in food induced by enzymes [7].

Longkong (*Aglaia dookkoo Griff*.) fruit is a non-climacteric tropical fruit native to Thailand and is widely cultivated in the southern part of Thailand, particularly in Narathiwat, Songkhla, Phatthalung, and Surat Thani provinces [8]. Longkong contains three parts: pericarp, flesh, and seeds, and among them, the pericarp is the major part, which contains an abundant level of polyphenolics. Studies have shown that longkong pericarp has numerous biological and pharmacological effects and is an essential alternative to synthetic agents. For example, Venkatachalam [9] reported that longkong pericarp extract (LPE) expressed a significant level of antioxidant, antimicrobial, anti-plasmodial, anti-proliferative, and anti-melanogenesis activities. In addition, LPE contains a rich source of lansic acid, lansiosides, lansiolic acid, lansiolic acid A, iso-onoceratriene, and could also control the hormonal imbalance in humans and promote anti-baldness, antipyretic, and anti-feeding activities. Furthermore, ellagic acid and corilagin in the LPE could promote anti-fibrosis and anti-glaucoma effects. Our previous study [10] showed that incorporating 1.5% LPE extract into the biodegradable, edible film significantly improved the film’s antioxidant and antimicrobial activities. In addition to the integration of plant extracts in the phyto-films, a recent advancement in the processing technologies shows that application of the sonication process, which is an inexpensive and reliable method, could enhance the physical barrier properties of the phyto-films. The process could also extract the phytochemicals from the plant ingredients and enhance their functional properties, mainly antioxidant and antimicrobial activities, by increasing the dispersion of macromolecules and phytochemicals all over the films.

Ultrasound treatment is one of the most common and prominent processing techniques, as it is straightforward and effective. It has been extensively employed in recent years in various sectors, including pharmaceuticals, phytochemical extraction, food, and many others. Sonication treatment might contribute to depolymerizing biopolymers by generating high pressure, resulting in the collapse of cavitational bubbles, which leads to the generation of intense heat in the residual bubbles and the development of mechanical force. Additionally, the mechanical force, cavitation effect, and shock waves generated by the sonication process may disintegrate biopolymers and phytochemicals, facilitating their dispersion and the complexation reaction between the extracted components and guest molecules [11]. Normally, the sonication process is controlled either by its amplitude or processing time. Gupta et al. [12] found that sonication could create the mechanical force and cavitational effect in the emulsion system at above 24 kHz power. Pratap-Singh et al. [13] reported that film emulsion treated at any fixed amplitude and increasing the processing time could effectively control particle size and improve the film. In this study, mung bean flour was incorporated with LPE and developed into an emulsion by processing it using ultrasonication at different time durations, and then the edible phyto-film was cast and checked for various physicochemical and functional properties.

## 2. Materials and Methods

### 2.1. Longkong Pericarp Extraction (LPE)

Longkong fruits were obtained locally from Surat Thani province, Thailand, and then they were thoroughly washed and any visible damages were removed and collected; the pericarp tissues were collected from the fruits and then processed into a powder and then extracted using the method of Nagarajan et al. [10]. First, 5 g of longkong pericarp powder was added into 20 mL 95% ethanol and extracted at 40 °C for 4 h using a water bath (W350, Memmert, Schwalbach, Germany) coupled with a vibrating shaker (210 Vib/min). After extraction, the mixture was covered and placed at ambient temperature overnight, filtered using a muslin cloth, followed by Whatman no. 1 filter paper. After that, the filtrate was collected and removed from the solvent using a rotary evaporator at 40 °C under low pressure. After completing the process, the LPE was collected and used in the film formation.

### 2.2. Film Formation

Mung bean films were prepared using a casting method. First, a 10% mung bean flour (*w*/*v*) was mixed with distilled water, and a slurry was prepared at 10,000 rpm for 10 min using a high-speed homogenizer. Then, the slurry pH was adjusted to 10 ± 0.02 by using NaOH (0.1M). After that, the slurry was gelatinized for 30 min by keeping it under the water bath, which was preset to 90 °C. Later, the slurry was removed from the water bath and cooled down to 40 °C in the ambient temperature while being stirred at a constant 500 rpm using a magnetic stirrer. Then, the slurry was incorporated with 2.5% glycerol (*w*/*v*), followed by 0.1% surfactant Tween 80 (*w*/*v*) and a 1.5% LPE. Then, the slurry was homogenized using a handheld ultrasonicator (Hielscher UP200Ht, Germany), in which an ultrasound probe was placed into the emulsion mix and run at 200 W power at 100% amplitude at different time durations (0 (C), 2 (T1), 4 (T2), 6 (T3), 8 (T4) and 10 (T5) min). Then, the slurry mixture was placed in the ultrasonication bath and treated at 25 kHz for 20 min to remove excess bubbles and then form-filmed. For film-forming, 15 mL of processed slurry solution was evenly poured into the Petri plates and dried at 40 °C in the hot air oven (Binder, Germany) for 24 h. After that, the plates were desiccated (50% relative humidity) for 24 h and prepared for various analyses.

### 2.3. Quality Analysis

#### 2.3.1. Physical Properties

The color characteristics of the film, such as lightness, redness, and yellowness, were randomly measured on the surface of the film using a handheld HunterLab colorimeter (Hunter Associates, Inc., Reston, VA, USA). The film thickness of the mung bean films was measured using a handheld digital micrometer (Mitutoyo 293-340-30 External Micrometer, Tokyo, Japan). For each treatment, ten films were used, and each film was tested for thickness at six random sites. The results were expressed in millimeters. The solubility of the films was tested based on the method of Aydogdu et al. [14]. Prior to the solubility test, the films were cut into squares (2.5 cm × 2.5 cm) and dried at 40 °C using a hot air oven for 24 h or until the films reached a constant weight (Wo). After that, the films were submerged in a beaker containing 50 mL distilled water for 24 h at an ambient temperature. After that, the film-dissolved solution was filtered and then dried in an oven at 105 °C for 24 h and the final weight (Wf) was recorded. Then, the following formula was used to calculate the solubility:

**Solubility (%) = [(Wo − Wf)/Wo] × 100**

The water vapor permeability of the films was measured based on the gravimetric modified cup method. First, the cylindrical test cups (40 mm internal diameter) were filled with 35 mL distilled water to facilitate the 100 % relative humidity in the cups. Next, the film thickness was randomly measured at six different spots on the film using a handheld digital micrometer (Mitutoyo 293-340-30 External Micrometer, Japan). Then, the films were set on the cups, and the caps were fitted to provide a taut surface and, after that, the cups were set aside to allow the water to permeate through the films. After that, the initial weight was recorded, and then the cups were placed in a pre-equilibrated desiccator (10–25% relative humidity) using silica gel. Then, the changes in the cup weight, relative humidity, and temperature in the desiccator were monitored and recorded every 2 h for 24 h. Finally, the following formula was used to calculate the water vapor permeability (WVP) of the film:

**WVP (Kg Pa^−1^ s^−1^ m^−1^) = (G × x)/t × A × S(R1 − R2)**

where G represented the water vapor flow (kg), x represented film thickness (m), t represented time (s), A represented area (m^2^), S represented saturated WVP (Pa) at measured temperature, R1 represented the relative humidity inside the cups, R2 represented the relative humidity inside the desiccator, and the X represented the multiplication.

#### 2.3.2. Mechanical Properties

Mechanical properties such as tensile strength, elongation at break (EAB), and Young’s modulus were measured in the films using the texture analyzer. Prior to analysis, the films were cut into small pieces (2 cm × 6 cm), and then the tensile strength was analyzed with 0.1 N preload at a fixed test speed of 0.30 mm/s. The measurement was ended when the film was torn apart into two pieces. The results of tensile strength were measured using the ratio of the maximum load to the cross-sectional area of the film. The results were expressed as MPa. EAB of the films was measured from the ratio between the total increment in film length at break and the film’s initial length (6 cm). The results were expressed as a percentage. Finally, the film’s Young modulus was measured using the stress–stress curve. The results were expressed as MPa.

#### 2.3.3. Phytochemicals and Antioxidant Activities

Films were extracted prior to phytochemical and antioxidant activities, 1 g of film samples was homogenized with 15 mL of 80% ethanol, and after that, the homogenate was centrifuged at 6000× *g* for 15 min. After centrifugation, a clear supernatant was collected and used for the following analysis.

For total phenolic content [15], 100 µL of WACV was added into the test tube and followed by the addition of 8.4 mL of distilled water and 500 µL of Folin–Ciocalteu reagent. After that, 1 mL of sodium carbonate (20%) was added and mixed well. Then, the reaction mixture was incubated at room temperatures for 30 min, and then it was measured under a spectrophotometer at 720 nm. The absorbance value of the sample was compared with the calibration curve made of gallic acid [10–100 µg/ mL; R2 value = 0.9970; *p* < 0.0001]. The results were expressed as µg gallic acid equivalence (GAE)/mL. For total flavonoid content [15], 250 µL of WACV was added into the test tube, and then 2.72 mL of 30% ethanol and 120 µL of 0.5 mol/L sodium nitrite were added and mixed well and kept for 5 min. Then, 120 µL of 0.3 mol/L aluminum chloride was added to the mixture, followed by adding 800 µL of 1 mol/L sodium hydroxide, and mixed well. After that, the reaction mixture was measured at 510 nm using a spectrophotometer. The absorbance value of the sample was compared with the calibration curve made of catechin [10–100 µg/ mL; R2 value = 0.9960; *p* < 0.0001]. The results were expressed as µg catechin equivalence (CHE)/mL.

For the DPPH (2,2-diphenyl-1-picrylhydrazyl) radical scavenging assay [16], 100 µL of WACV was added into a test tube that contained 3.9 mL of 60 µmol/L DPPH and mixed well. After that, the reaction mixture was incubated for 30 min in a dark condition at ambient temperature. Then, the reaction mixture was measured at 515 nm using a spectrophotometer. The results were expressed as a percentage of DPPH radical scavenging ability. For ABTS (2,2′-azino-di-(3-ethylbenzthiazoline sulfonic acid radical cation scavenging assay), a 100 µL of WACV was added to a test tube and mixed with 100 µL ABTS reagent (as described in Lee et al. [17]) in a 96-well microplate, and then it was incubated for 6 min at room temperature. After incubation, the sample was measured at 734 nm using a microplate. The results were expressed as a percentage of ABTS radical scavenging ability. For FRAP (ferric reducing potential antioxidant assay), 100 µL of WACV was mixed with 3 mL of FRAP reagent (as described in Alberti et al. [15]). The reaction mixture was incubated for 20 min to develop a blue color complex, and after that, it was measured at 593 nm using a spectrophotometer. The results were expressed as a percentage of ferric-reducing antioxidant potential. For the hydroxyl radical scavenging assay [18], 1 mL of WACV was added in a test tube that containing 1 mL of ferrous ammonium sulfate (0.13%-Ethylenediamine tetra acetic acid (EDTA) (0.26%) solution, 0.5 mL of 0.018% EDTA, 1 mL of 0.85% dimethyl sulfoxide, and 0.22% ascorbic acid. After that, the reaction mixture was mixed well and incubated in a water bath at 90 °C for 10 min. Then, the reaction was terminated by adding 1 mL of ice-cold trichloroacetic acid (TCA). After that, 3 mL of Nash reagent wax was mixed in the reaction mixture and kept at room temperature for 15 min to develop yellow color. Then, the reaction mixture was measured at 412 nm using a spectrophotometer. The results were expressed as a percentage of hydroxyl radical scavenging ability.

#### 2.3.4. Antimicrobial Activity

The antimicrobial activity [19] of the films was tested against the two groups of microorganisms, which are Gram-positive (*L. monocytogens*, *S. aureus*, and *B. subtilis*) and Gram-negative (*C.jejuni*, *E. coli*, and *P. aeruginosa*). A film disc (1.5 cm diameter) was submerged in a glass tube that contained 10 mL of tryptic soy broth (TSB) and brain heart infusion broth (BHIB) medium. After that, 1 mL of Gram (+) (in TSB medium) and Gram (-) (in BHIB medium) bacteria was added and mixed. Then, the tubes with a medium that contained *L. monocytogens*, *S. aureus*, and *B. subtilis* were transferred to the shaker and shaken at 200 rpm at room temperature for 24 h, whereas the tubes that contained *C. jejuni*, *E. coli*, and *P. aeruginosa* were incubated for 48 h at 37 °C with microaerophilic conditions. After incubation, 1 mL of microbial aliquots from each tube was collected and tested for its optical density (OD) at 600 nm (*L. monocytogens* (0.418 OD_600_), *S. aureus* (0.782 OD_600_), *B. subtilis* (0.893 OD_600_), *C. jejuni* (0.613 OD_600_), *E. coli* (0.597 OD_600_), and *P. aeruginosa* (0.657 OD_600_)) and then serial diluted using buffered peptone water. Then, Gram-positive bacteria was plated on the Mueller-Hinton agar plates and incubated for 24 h at 37 °C, whereas Gram-negative bacteria such as *C. jejuni* was plated on Campylobacter agar plates and other bacteria in this group was plated on MacConkey agar and incubated for 48 h at 37 °C. After incubation, the plates for the count for colonies and the results were expressed as log CFU/mL.

### 2.4. Statistical Analysis

All the data were represented as mean ± standard deviation (*n* = 3). The statistical analysis of the data was carried out using the Statistical Package for the Social Sciences (SPSS) software version 12 for Windows. The significant differences (*p* < 0.05) among the mean values of the measurements were carried out by using the analysis of variance (ANOVA) and followed by Tukey’s Multiple Comparison Test.

## 3. Results and Discussion

### 3.1. Color Characteristics

**Figure 1:**
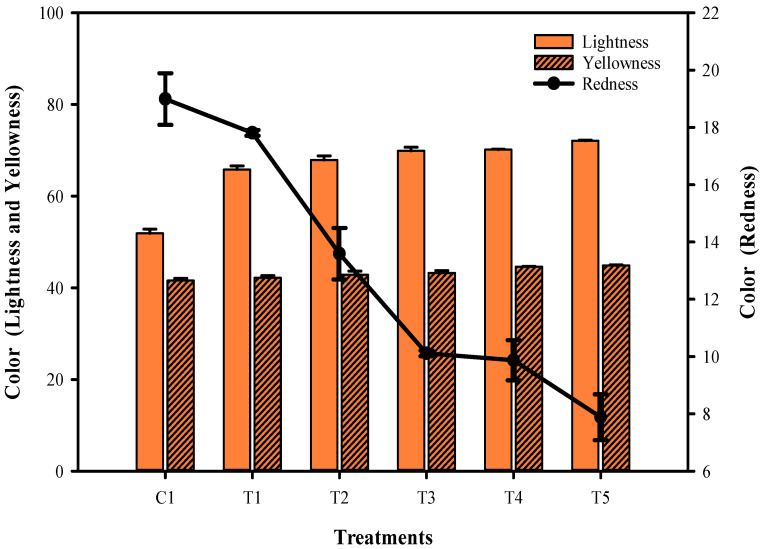
Color characteristics (lightness, yellowness, and redness) of the mung bean phyto-film that incorporated LPE and was processed under sonication.

The changes in color characteristics of the phyto-films, which are made of mung bean flour and combined with LPE and sonicated at different times, are shown in Figure 1. The results indicated that the time differences in the sonication process had significantly influenced the color and transparency of the films. Although a clear trend in the color of the phyto-films was observed, an increase in the duration of the sonication process had significantly increased the lightness and yellowness of the phyto-films, and on the other hand, the redness of the films was drastically decreased (*p* < 0.05). Color is essential for plant and food-related films, since it directly impacts consumers’ perception [20]. The mung bean flour is typically bright yellowish, while the LPE is slightly drab, and when the sonication treatment was performed to the film mixture, the method dramatically impacted this color combination, brightening the phyto-film color and making it more yellowish. According to Gomes et al. [21], sonication on chitosan-based edible films substantially enhanced color opaqueness and adversely affected the yellowness and redness due to the lack of impact of light transmission on the film. In another investigation, Tavares et al. [22] found that the interaction rate of protein and polysaccharide contents in edible composite phyto-films was the primary cause of color change. Beikzadeh et al. [23] found that prolonged sonication could break down the polysaccharide chains and induce a new chemical bond, and therefore, a denser film network formed. Ji et al. [24] reported that the extended sonication time had increased the composite film’s color and transmittance.

### 3.2. Film Thickness, Solubility, EAB, TS, Young’s Modulus, and WVP

**Figure 2:**
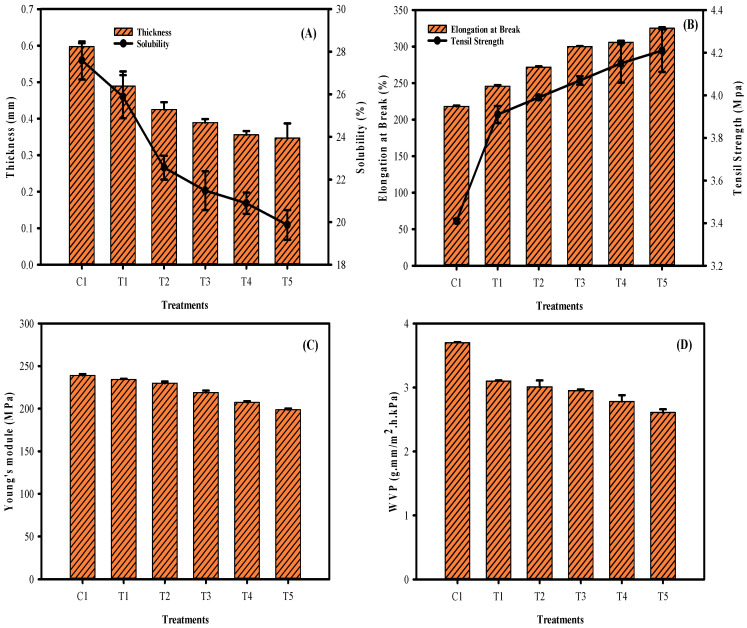
Thickness (**A**), solubility (**A**), elongation at break (**B**), tensile strength (**B**), Young’s modulus (**C**), and water vapor pressure (**D**) of the mung bean phyto-film that incorporated LPE and was processed under sonication.

The film thickness of the phyto-films made of mung bean flour and LPE and sonicated at different times are shown in Figure 2A. Typically, film thickness plays a vital role in accessing the mechanical and permeability properties of the film [25]. The results showed a significant change in the film thickness. A continuous increment in the sonication period had significantly thinned the film thickness. Sonication had affected thickness when treated for 2–6 min, and afterward, the effect was not much different (8–10 min). In addition to sonication, adding phenolic sources could also adversely affect the film’s thickness. The present findings are in accordance with the study of Arcan and Yemenicioglu [26]. Similarly, the solubility of the films was continuously decreased (*p* < 0.05) with the extended sonication process, indicating that the hydrophobicity of the film is continuously increased with the sonication process (Figure 2A). Mangmee and Homthawornchoo [27] reported that adding plant extract in the edible film could decrease water solubility. It might be due to the formation of intermolecular hydrogen bonding among the hydroxyl group of polysaccharides and proteins, which thus decreased the film’s affinity towards water. Wang et al. [28] mentioned that protein in the edible film also contributes to poor solubility. Furthermore, extended sonication could induce aggregation of protein and lipoproteins, and as a result, hydrophobicity drastically increased and thus reduced the water solubility. Figure 2B shows the elongation at break and tensile strength of the film. The results showed that the extended sonication treatment had significantly increased the flexibility of the film. A decrease in thickness is intercorrelated with the increased flexibility of the films tested. Similarly, the sonication process contributed to the increased tensile strength in the tested films. The pronged duration of sonication had significantly increased the tensile strength compared with the non-sonicated samples. Sonication treatment could enhance ghost destruction and release the amylose contents, a key ingredient for improving the tensile strength in the phyto-films [29]. Typically, Young’s modulus (YM) represents the rigidity of the film; the higher the YM value means, the higher rigidity it refers to. This study results found that phyto-film emulsion treated with sonication for a prolonged time significantly decreased the YM values when compared with the control samples, indicating the flexible film formation (Figure 2C). Significant differences were noted between T3–T5 samples; however, the T1 and T3 samples did not differ much. On the other hand, the YM values were predominant in the control sample, and consequently, a stiffer film property was observed. Water vapor permeability (WVP) represents the moisture barrier property of the films, and it should be as small as possible to control the moisture transmission between the phyto-film and surrounding environment [30]. WVP level in the tested film showed that prolonged sonication significantly controlled the WVP rate as compared with the control (Figure 2D). Extended sonication duration was more prominent in controlling WVP than fewer sonication treatments. WVP results are in accordance with the solubility results, as the prolonged sonication time effectively controlled solubility, thus increasing the films’ hydrophobicity, which could decrease the WVP rate. Furthermore, the sonication process could expel the phenolic compounds from the LPE extract in the film, thus reducing the void space in the film matrix and possibly controlling the penetration of water vapor [31,32]. Beikzadeh et al. [23] suggested that a decrease in WVP in the phyto-films by ultrasound could be due to the development of covalent bonding between vapor, water molecules, and sonication-generated free radicals. This could trap the water molecule within the film matrix and thus reduce the WVP, and this effect increases with the duration of sonication as it generates more weak free radicals.

### 3.3. Film Droplet Size, Polydispersity Index, and Zeta Potential

**Figure 3:**
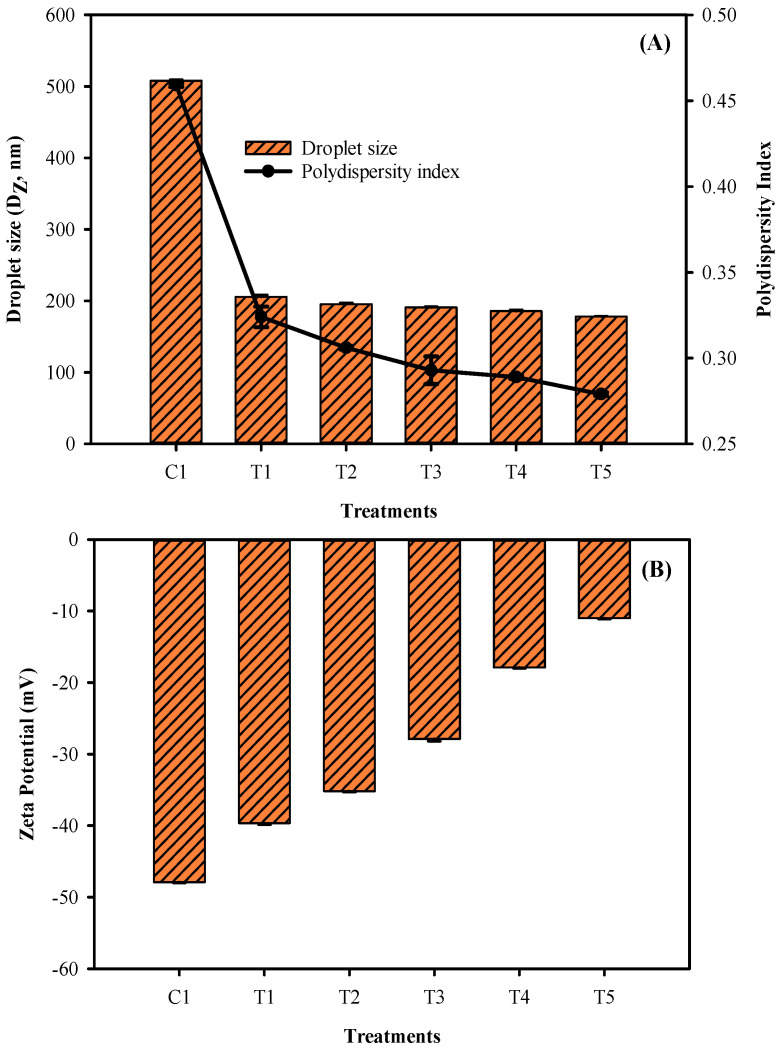
Droplet size (**A**), polydispersity index (**A**) and zeta potential (**B**) of the mung bean phyto-film that incorporated LPE and was processed under sonication.

The droplet size and polydispersity index of the phyto-films made of mung bean flour and LPE and sonicated at different times are shown in Figure 3A. As the sonication time in the film emulsion was prolonged, the droplet size and polydispersity index of the films decreased. Compared to the films that employed sonication, the control group showed larger droplet size and polydispersity. However, according to the findings, there were no considerable differences in film droplet results across treatments. It appears that shorter (T1) and more extended (T5) periods of sonication have an impact on the film’s droplet size, whereas the most negligible differences (T2–T4) in duration had very little impact on the droplet size and polydispersity index. This could be due to the over-processing effect, or to the fact that the minimum sonication duration is sufficient to create a nano-emulsion, requiring more extended periods of sonication treatment to break the film emulsion further. Furthermore, the energy supplied to the film emulsion during processing may cause a reduction in droplet size in phyto-films treated with ultrasonication. The presence of surface charge results in a zeta potential, which may provide information on electrical interaction forces among the distributed particles. Zeta potential is defined as the electrokinetic potential at the boundary of the hydrodynamic shear plane of a charged particle adjacent to a solid surface exposed to a liquid. The zeta potential results showed that electric charges on the phyto-film samples were significantly higher in the control samples, and with the sonication process, it gradually decreased as the treatment duration increased (*p* < 0.05). It indicates that the sonication process somehow adversely affected the electrostatic repulsion of the film and adversely affected the stability of the colloidal system of film. Yin et al. [33] reported that phyto-film with the colloidal system for maintaining good physical and barrier properties and preventing coalescence requires a minimum zeta potential value of ±30 mV to generate sufficient electrostatic repulsion. This study’s results showed that when the phyto-films were treated with sonication duration longer than 6 min, it adversely decreased the zeta potential value; however, it is still higher than the minimum required range, indicating the suitable physical property of the films.

### 3.4. Phytochemicals and Antioxidant Activities

**Figure 4:**
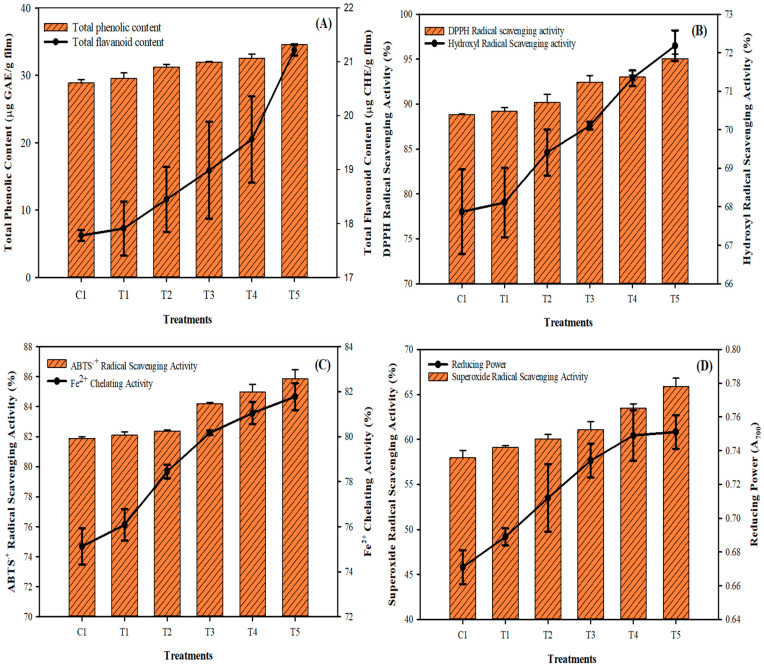
Phytochemical (**A**) and radical scavenging abilities (**B**–**D**) of the mung bean phyto-film that incorporated LPE and was processed under sonication.

The phytochemicals, such as TPC and TFC, in the phyto-films are made of mung bean flour emulsion and combined with LPE and sonicated at different times. They are shown in Figure 4A,B. The results showed the TPC and TFC contents were naturally higher in the films, which could be due to the richness of phytochemical, which is contributed by the mung bean flour and LPE [4,8]. The sonication process improved the treated sample’s TPC and TFC contents, and the levels gradually increased with the sonication process. Ma et al. [34] also observed an elevated level of polyphenolics in the edible films treated with ultrasonication at various intervals. Muñiz-Márquez et al. [35] reported that sonication time and extraction solvents played a significant role in extracting phytochemicals, and they also mentioned that the extended sonication time had extracted different phenolics. The results found that TPC is higher than the TFC in the films, indicating that other phenolic compounds are higher in the phyto-films, followed by flavonoids. It could be due to the addition of LPE, which contains more phenols, lignin, and flavonoids [9]. The study of Karabegović et al. [36] observed that the ultrasonication process could extract more phenolics than flavonoids. The antioxidant activities (DPPH radical, hydroxyl radical, ABTS radical, superoxide radical scavenging activities, and Fe^2+^ chelating activity and reducing power) of the mung bean phyto-films are shown in Figure 4C,D. Overall, the antioxidant activities in the films were continuously increased with the sonication times. Among the various antioxidant activities, the films showed potent scavenging against the DPPH and ABTS radicals, followed by hydroxyl and superoxide radicals. The sonication process significantly increased the films’ radical scavenging activity, and the prolonged treatment duration showed better performance. Furthermore, the increasing reducing power values of the phyto-films indicate an excellent donor of electrons, which could easily reduce the radicals by scavenging. The antioxidant potency of the mung bean phyto-films is directly in accordance with the results of TPC and TFC, which are the key components supporting antioxidant properties in plants. Cao et al. [37] reported that mung bean flavonoids exhibited strong antioxidant activities against the DPPH, hydroxyl and superoxide radicals. Aparicio-Fernández et al. [38] observed that phyto-film added to red prickly pear extract had significantly increased the antioxidant potency of the films and is mainly ascribed to the presence of polyphenolics. This study found that phyto-film emulsion treated with sonication had significantly increased the antioxidant activity compared to control. The ultrasonication process increases the phyto-film’s surface area and thus induces the phytochemical in the films to provide antioxidant properties. It could reduce the lipid particle size, which generally interrupts the film emulsion’s biopolymers and decreases the surface area [39,40].

### 3.5. Antimicrobial Activities

**Figure 5:**
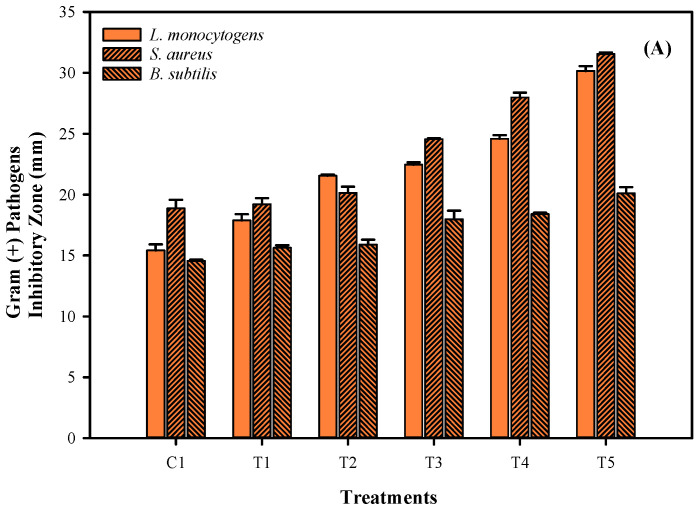

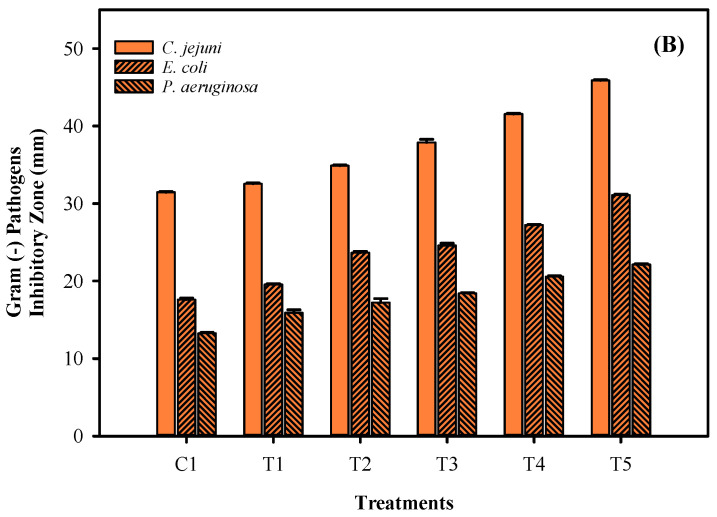
Antimicrobial activities (gram (+) pathogen inhibition (**A**) and gram (−) pathogen inhibition (**B**)) of the mung bean phyto-film incorporated with LPE and was processed under sonication.

Antimicrobial activities of phyto-films made of mung bean flour emulsion and combined with LPE and sonicated at different times are shown in Figure 5. This study tested film antimicrobial activity against both selected Gram (+) and Gram (-) pathogenic bacteria. Overall, the results showed a significant control against the pathogenic bacteria by the phyto-films. Gram (-) bacteria are most affected by the tested film’s antimicrobial activity, followed by the Gram (+) bacteria. Gram (-) bacteria such as *C. jejuni* and *E.coli* were significantly reduced by the films, followed by the *P. aeruginosa*. Similarly, the gram (+) bacteria, including *S. aureus* and *L. monocytogens*, were effectively controlled by the phyto-films, followed by the *B. subtilis*. A significant level of control against the pathogenic bacteria was observed in control and sonicated phyto-films. Mung bean and LPE have been widely reported for their antimicrobial capacity. Mung bean contains an abundant level of biocides in the form of phytochemicals and thus primarily acts against the microbial cell membrane by disrupting their proton motive force, electron flow, active transport mechanism, and coagulation of cell compositions [41,42]. Furthermore, longkong pericarp is highly sensitive to any form of external stress and thus generates abundant levels of lignin by activating polyphenol oxidase enzymes [43]. Lignin, as a polyphenolic, acts as a potential antimicrobial agent by attaching to the bacterial membrane, generating localized heat, and creating a reactive oxygen species, which allows the light irradiation to destroy the microbial membranes [44]. Additionally, the sonicated films had also significantly increased the antimicrobial activity against the studied pathogens when treated with the sonication at extended times. Generally, the phyto components are excellent in controlling the pathogenic and food-spoilage bacteria [45]. The extended period of sonication could increase the micro-cavitation of the film and thus immobilize the LPE into the pores and consequently produce increased antimicrobial activity. A similar finding was observed in Wang et al. [46], and they reported that increased sonication time had significantly increased the immobilization process. In another study, Ma et al. [47] observed that the application of sonication could loosen up the film and promote the release of incorporated antimicrobial compounds and establish antimicrobial activity. Liu et al. [48] studied the sonication process on the composite film, and their observation found that sonication increased the TPC level in the film and thus effectively controlled *S. aureus* and *E.coli*.

## 4. Conclusions

The development of antioxidant and antimicrobial films using mung bean flour and longkong pericarp extract, assisted with sonication at different time durations, was studied in this work. Sonication at different time periods positively influenced the phyto-film and improved various physico–chemical, antioxidant, and antimicrobial activities. The addition of longkong pericarp extract into the mung bean flour emulsion significantly increased the antimicrobial and antioxidant activities of the film. The phyto-film emulsion, when sonicated at different times, helped immobilization of the longkong pericarp extract in the film pores and thus promoted the functional property of the film. Sonication treatment at a prolonged period caused the thickness, solubility, and water vapor permeability of the phyto-film to be decreased. The sonicated phyto-film emulsion heavily decreased its droplet size below 200 nm and changed into a nano-emulsion. The prolonged duration of sonication maintained the zeta potential value below −30 mV, making it a strong and stable film. The prolonged sonication effectively increased the antioxidant and antimicrobial functionality of the film and made it a suitable candidate for applications in the food and drug industries. The present study recommends that phyto-film emulsion sonicated at 6–8 min before casting it into film could be an excellent processing technique that widely improves the film’s functionality in a variety of ways.

## Data Availability

Not applicable.
